# Are Tip–Apex Distance and Surgical Delay Associated with Increased Risk of Complications and Mortality Within the First Two Years After Surgery for Femoral Neck Fractures?

**DOI:** 10.3390/jcm14144991

**Published:** 2025-07-15

**Authors:** Jacob Schade Engbjerg, Rune Dall Jensen, Michael Tjørnild, Daniel Wæver, Rikke Thorninger, Jan Duedal Rölfing

**Affiliations:** 1 Department of Orthopaedics, Regional Hospital Randers, Skovlyvej 15, 8930 Randers, Denmark; michael.tjornild@rm.dk (M.T.); danwae@rm.dk (D.W.); 2 MidtSim, Department of Clinical Medicine, Aarhus University, Central Denmark Region, Hedeager 5, 8200 Aarhus N, Denmark; rune.dall@rm.dk (R.D.J.); jan.rolfing@rm.dk (J.D.R.); 3 Department of Clinical Medicine, HEALTH, Aarhus University, Palle Juul-Jensens Boulevard 11, 8200 Aarhus N, Denmark; 4 Department of Orthopaedics, Aarhus University Hospital, Palle Juul-Jensens Boulevard 99, 8200 Aarhus N, Denmark; rikkthor@rm.dk

**Keywords:** femoral neck fractures, dynamic hip screw, tip-apex distance, surgical delay, two-year complication rates, two-year mortality rates

## Abstract

**Background:** Femoral neck fractures (FNF) have high mortality rates. Surgical delay likely contributes. Dynamic hip screw (DHS) fixation is common for FNF, and the tip–apex distance (TAD) measures screw position in the femoral head. A TAD >20–25 mm is considered a risk factor for screw cut-out. This study investigated the association between (1) surgical delay and complication/mortality rates within two years post-surgery and (2) TAD and postoperative complications following DHS in FNF. **Methods:** This retrospective study included 325 FNF patients who underwent DHS osteosynthesis at Regional Hospital Randers, Denmark from 2015–2021. The primary composite outcome included complications measured on radiographs (e.g., screw cut-out, avascular necrosis), reoperation, or death within 2 years. Radiographs were evaluated for TAD and postoperative complications/reoperations. Surgical delay was defined as the time from the diagnostic radiograph to the start of the operation. TAD was measured based on radiographs. Electronic patient journals and national SSN registers were used to assess reoperation and death rates. **Results:** The mortality rate was 16% within 1 year and 26% within 2 years. The complication rate was 16% within 2 years. The median surgical delay was 7.9 h (IQR 5; 14). Surgical delay was significantly associated with the 1-year mortality rate (*p* < 0.01), but not with the 1-year complication rate (*p* = 0.77). Surgical delay was associated with complication rates 2 years post-surgery only for undisplaced fractures, *p* = 0.046. The median TAD was 16.3 mm (13.8; 18.7); no association with complications <1 year (*p* = 0.56) or <2 years (*p* = 0.99) was observed. There were 59/325 patients with TAD >20 mm, six of whom had complications, and 6/59 with TAD >25 mm, none of whom had complications. **Conclusions:** We report a significant association between surgical delay and mortality rates in FNF, despite surgical delays <24 h. Additionally, there was a significant association between surgical delay and risk of complication/reoperation 2 years post-surgery for undisplaced fractures. No association was found between TAD and complication rates following DHS fixation for FNF; however, only a few TAD outliers were observed.

## 1. Introduction

Hip fractures (HF) are a common orthopedic injury, and although incidence rates have declined in recent years, they are expected to rise in the coming decades due to demographic changes [1,2,3,4,5]. HF are associated with increased morbidity and are responsible for the majority of fracture-related mortality in the population over 50 years [6,7,8]. Mortality rates following HF are well-documented, and the 1-year mortality rate has been reported to be up to 30% [9,10,11,12,13]. Surgical delay, defined as the time from radiological diagnosis to surgery, may negatively impact mortality rates. Consequently, several studies and guidelines recommend surgery within 48 h of diagnosis [14,15,16,17]. However, conflicting literature exists on the effect of accelerated surgery within 24 h from HF diagnosis on 30-day and 1-year mortality rates [18,19,20,21,22,23]. Similarly, little is known about the impact of surgical delay on 2-year mortality rates.

Reoperations after HF surgery are associated with increased morbidity and mortality [24,25,26]. When a HF is managed with internal fixation with a dynamic hip screw (DHS), a common complication is cut-out of the lag screw through the femoral head [27]. A tip-apex distance >25 mm was shown by Baumgaertner et al., to increase the risk of cut-out significantly [28]. Following this, the implant position assessed by the TAD >25 mm was established as a strong predictor for cut-out [28]. Since TAD was introduced in 1995 [28], the significance of 25 mm as a cut-off for increased risk of cut-out in trochanteric fractures has been debated [27,29]. The literature on the association between TAD and the risk of cut-out in femoral neck fractures (FNF) is sparse.

We, therefore, aim to investigate whether surgical delay is associated with mortality rates 1 and 2 years after DHS surgery in FNF. Secondly, we aimed to examine the association between TAD and the risk of complications/reoperation in FNF patients.

## 2. Materials and Methods

A retrospective study of FNF patients, Garden type 1–4 [30], operated with DHS at Regional Hospital Randers, Denmark with a minimum 2-year follow-up, was conducted.

Inclusion criteria: FNF from 2015 to 2021 operated with 2–4-hole DHS and available intraoperative radiographs to evaluate TAD.

Exclusion criteria: FNF operated with three cannulated screws, hemi- or total hip-arthroplasty, inter- and sub-trochanteric fractures misclassified as FNF, other fractures and procedural codes misclassified as FNF, a combination of DHS and cannulated screws, no available intraoperative radiographs, and 7-hole DHS and intramedullary nailing for FNF.

Data collection: Patients were identified using the Central Denmark Region’s Business Intelligence-portal, and a search on diagnosis code DS720 (FNF) was conducted.

All preoperative radiographs were evaluated to confirm FNF type, and all intraoperative radiographs were assessed to verify operation type (2–4 holes DHS).

### 2.1. Surgical Delay and Other Data Sources

Surgical delay was defined as the time from the primary radiograph diagnosing the FNF to the time of skin incision (operation start).

Information on operation start and operation time was accessed in the electronic patient file system (EPJ) in the Central Denmark Region Business Intelligence portal.

Mortality data were found in the Data Warehouse of the Central Denmark Region and the Danish SSN register.

Comorbidity was assessed with the Charlson Comorbidity Index (CCI).

ICD-10 diagnosis codes were extracted from EPJ in the Central Denmark Region and used to calculate the CCI score.

### 2.2. Evaluation of Radiographs

All pre-, peri-, and postoperative radiographs were evaluated using the local Picture Archive and Communication System (PACS). All radiographic evaluations were performed by co-author JSE using a standardized method, as described by Baumgaertner et al. [28].

Fracture classification: FNF were classified according to the commonly used [31] Garden Classification using the anterior–posterior plane from the preoperative radiograph [30]. Fracture types 1 and 2 are undisplaced fractures, and fracture types 3 and 4 are displaced fractures.

**Figure 1:**
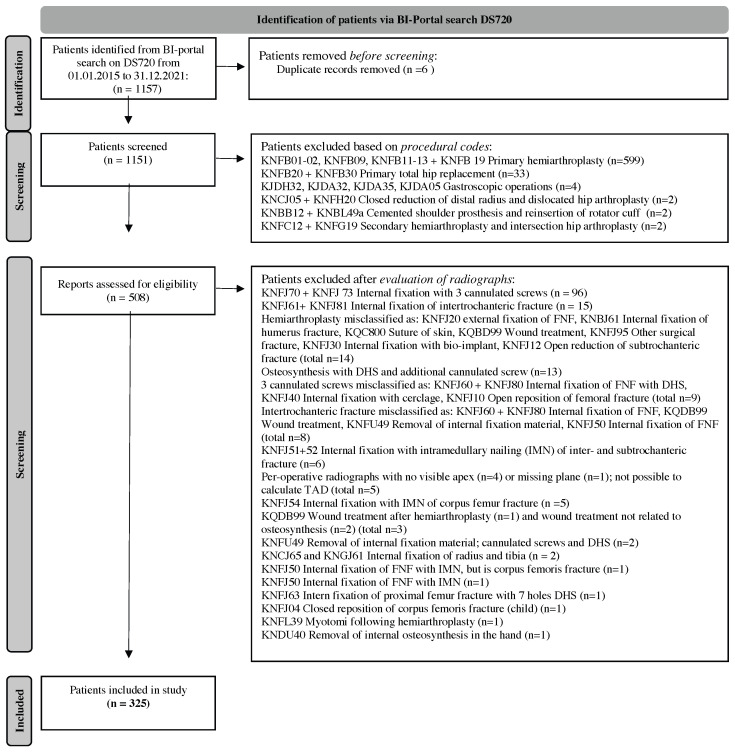
Flowchart showing identification of eligible patients for inclusion in the study. A total of 325 patients were included in the study. FNF = femoral neck fractures; DHS = dynamic hip screw; IMN = intramedullary nailing.

Tip–apex distance (TAD): TAD was measured on the intraoperative radiograph (imaging documenting the osteosynthesis) according to Baumgaertner et al. [28]. The radiograph was corrected for magnification by measuring the diameter of the lag screw on the radiograph and dividing it by the known diameter of the screw (12.5 mm) (For equation see “Figure 1” in [28]).

Implant position: The femoral head was divided into nine zones according to Cleveland et al. and Kyle et al. [32,33]. The placement of the screw in the femoral head was evaluated in both anterior–posterior and axial intraoperative radiographs, and the placement of the tip of the screw was noted.

### 2.3. Complications/Reoperation

All radiographs taken within 2 years from the primary operation were evaluated for complications or reoperation, for example, hemiarthroplasty or total arthroplasty. Complications were defined as cut-out of the lag screw in the femoral head, necrosis of the femoral head, and non-union, regardless of whether the patient received a reoperation or was treated conservatively.

### 2.4. Statistics

All calculations were made using Stata, StataCorp. 2023. Stata Statistical Software: Release 18. College Station, TX: StataCorp LLC.

Data are presented as medians with interquartile ranges (IQR) due to non-Gaussian distribution.

Categorical variables, such as fracture type (undisplaced vs. displaced) and complication/reoperation rates, were compared using the chi-squared test. When expected cell counts were below five, Fisher’s exact test was applied to ensure statistical validity. For all additional associations, e.g., the analysis of the effect of surgical delay on mortality and complication rates and the effect of TAD on mortality and complication rates, the Mann-Whitney U test was used, as these variables did not meet the assumptions of normality.

The association between lag screw placement zones and complication/reoperation rates was analyzed using central–central placement as the reference category.

To account for potential confounding factors, a multivariable logistic regression analysis was performed. Variables included in the model—age, Charlson Comorbidity Index (CCI) score, sex, and surgical delay—were selected based on their significant univariate association with mortality. This approach allowed for the adjustment of confounding effects on mortality outcomes. 

A *p*-value < 0.05 was considered statistically significant for all tests.

### 2.5. Language

Language and readability improvements were supported by the use of AI-based writing assistance using Microsoft Copilot, GPT-4. After using this tool, the authors reviewed and edited the content as needed and take full responsibility for the content of the publication.

## 3. Results

### 3.1. Inclusion

A total of 1157 patients were identified based on the FNF diagnosis code DS720 and were eligible for inclusion. After assessing eligibility based on inclusion and exclusion criteria, 325 patients were included in the final analysis.

Exclusion most frequently occurred due to the following three reasons: operation with primary hemiarthroplasty (n = 599), internal fixation with three cannulated screws (n = 96), and primary total hip replacement (n = 33).

Please refer to Figure 1 for a detailed flowchart of the inclusion of patients.

### 3.2. Surgical Delay

The median surgical delay for all patients during the study period was 7.9 h (5; 14). Of the 325 patients included data on operation start existed for 316, with 303 of these (96%) being operated on within 24 h of the time point of radiological diagnosis.

Surgical delay was significantly associated with the one-year mortality (*p* < 0.01): 10.9 h (7; 17) for patients deceased <1 year and 7.5 h (5; 14) for patients still alive. This finding was still significant after two years (*p* < 0.01): 10.3 h (6.4; 15.2) for patients deceased <2 years and 7.5 h (4.6; 13.8) for patients still alive (Table 1).

After adjusting for the effect of CCI score and age using multivariate regression analysis, the surgical delay was still significantly correlated with the one-year mortality, but not two-year mortality, as described below.

No effect of surgical delay on the risk of complication/reoperation was found for all fracture types (displaced/undisplaced) analyzed together. A subgroup analysis of undisplaced and displaced fractures showed a significant association between surgical delay and the risk of complications/reoperation within two years for undisplaced fractures (*p* ≈ 0.046), but interestingly not for displaced fractures (*p* = 0.31; Table 2).

### 3.3. Mortality

An overall one-year mortality rate of 16% and a two-year mortality rate of 26% were observed during the study period (Table 3).

### 3.4. Implant Placement According to Tip–Apex Distance (TAD)

The overall median TAD was 16.3 mm (13.8; 18.7), with no statistically significant difference (*p* = 0.56) between patients with and without complications/reoperation <1 year: TAD 16.3 mm (13.7; 18.7) vs. 16.7 mm (14.1; 19.2). No statistically significant difference was found between patients with and without complications/reoperation <2 years (*p* = 0.99): TAD 16.3 mm (IQR 13.7; 18.7) vs. 16.6 mm (IQR 14; 18.5) (Table 1).

**Figure 2:**
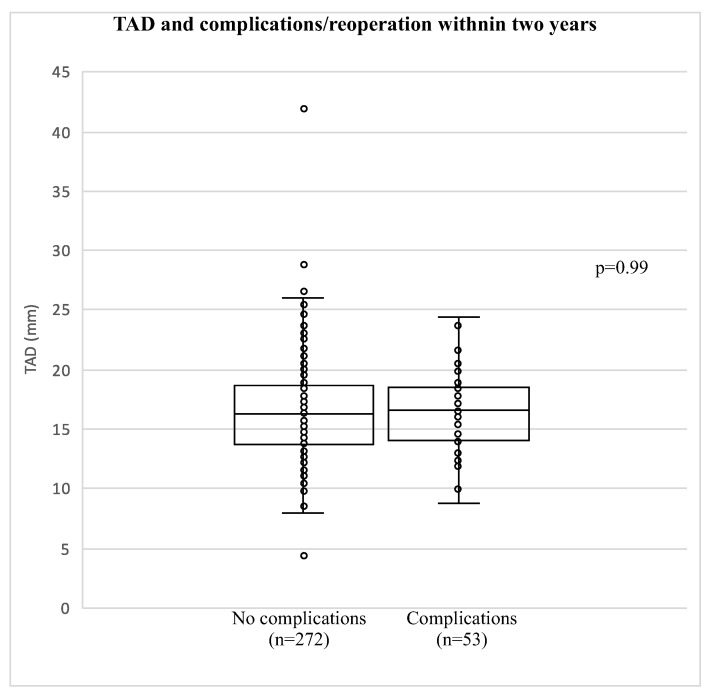
Tip–apex distance and risk of complications/reoperation. Box plot illustrating the distribution of TAD based on complications/reoperations. The box shows the median, 25th percentile, and 75th percentile. (**Left**): no complications or reoperations <2 y. (**Right**): complications or reoperations <2 y. As seen, no TADs >25 mm were associated with complications, and only six patients with a TAD between 20 mm and 25 mm experienced complications/reoperation. TAD outliers >20 mm were not associated with an increased risk of complications/reoperation.

No association between TAD and the 1-year mortality rate was found (*p* = 0.6), and no association with the 2-year mortality rate was found (*p* = 0.6) (Table 1). Notably, there were 59/325 cases with TAD >20 mm and only 6/59 with TAD >25 mm in total (Figure 2). None of the TAD outliers >25 mm resulted in complications/reoperation, and only six cases with a TAD between 20 mm and 25 mm resulted in complications/reoperation.

### 3.5. Implant Placement According to Cleveland Zones

**Figure 3:**
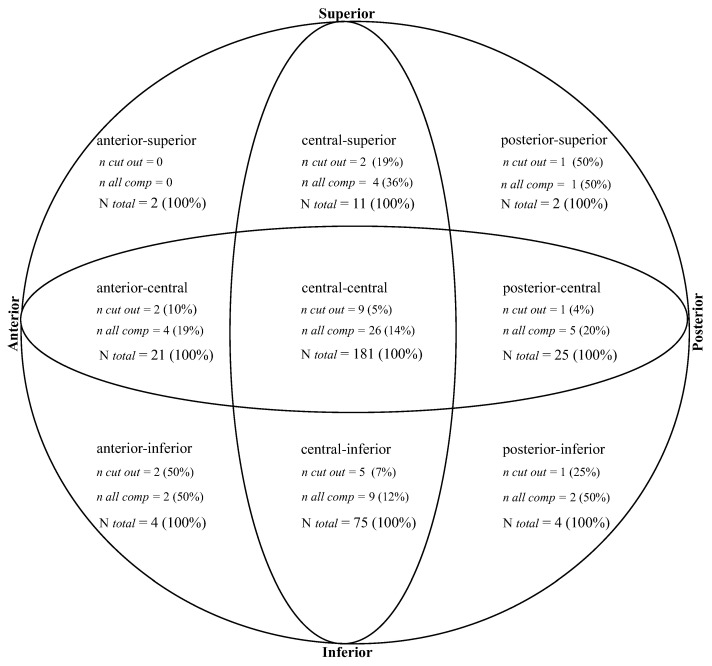
Screw placement in the femoral head. The femoral head was divided into 9 zones based on AP and axial radiographs. “n cut out” represents the number of screw cut-outs from the zone within 2 years from DHS surgery, with the cut-out frequency in parentheses. “n all comp” represents all complications from the zone including the following: cut out, necrosis of the femoral head, non-union, and conversion to THA within 2 years from surgery. The total complication frequency is shown in parentheses. “n total” represents the total number of screws placed in the zone. The majority of screws were placed center-center. A trend toward superior–central having more cut-outs than central–central and central–inferior placement, and anterior–central having more cut-outs than posterior–central placement was observed. However, these trends were non-significant, as no significant difference in cut-out frequency or complication frequency between zones was found.

Of the 325 implants, 181 (56%) were placed central–central in both planes (Figure 3). Within two years, 26 patients (14%) with central-central placed implants experienced complications or required reoperation, with a cut-out rate of 5%.

Central–inferior: 75/325 (23%) implants. Nine of these 75 patients experienced complications/reoperation within two years (12%). The complication/reoperation rate was not statistically significantly different from that of central–central placement, *p* = 0.77 (Chi-square). A cut-out rate of 5/75 (7%) was found.

Central–superior: 11/325 (3%) implants, of which 4/11 (36%) experienced complications/reoperation within two years. This rate was not statistically significantly different from that of central–central placement, *p* = 0.11 (Fisher’s exact test). A cut-out rate of 2/11 (19%) was found.

Anterior–central: 21/325 (6.5%) implants, of which 4/21 (19%) experienced complications/reoperation within two years. The complication/reoperation rate was not statistically significantly different from that of central–central placement, *p* = 0.75 (Fisher’s exact test). A cut-out rate of 2/21 (10%) was found.

Posterior–central: 25/325 (8%) implants, where 5/25 (20%) had complications/reoperation within two years. The complication/reoperation rate was not statistically significantly different from that of central–central placement, *p* = 0.56 (Fisher’s exact test). A cut-out rate of 1/25 (4%) was found.

Anterior–inferior: 4/325 (1.2%) implants, where 2/4 (50%) had complications/reoperation within two years. The complication/reoperation rate was not statistically significantly different from that of central–central placement, *p* = 0.12 (Fisher’s exact test). A cut-out rate of 2/4 (50%) was found.

Posterior–inferior: 4/325 (1.2%) implants, where 2/4 (50%) had complications/reoperation within two years. The complication/reoperation rate was not statistically significantly different from that of central–central placement, *p* = 0.12 (Fisher’s exact test). A cut-out rate of 1/4 (25%) was found.

Anterior–superior: 2/325 (0.6%) implants. 0 had complications/reoperation within two years. The complication/reoperation rate was not statistically significantly different from that of central–central placement, *p* = 1 (Fisher’s exact test).

Posterior–superior: 2/325 (0.6%) implants. 1/2 (50%) had complications/reoperation within two years. The complication/reoperation rate was not statistically significantly different from that of central–central placement, *p* = 0.28 (Fisher’s exact test). A cut-out rate of 1/2 (50%) was found.

### 3.6. Complications

A total of 47 of 325 patients (15%) experienced complications/reoperation within one year. An additional six patients had complications/reoperation after one year, but within two years from primary surgery, resulting in a total of 53 of 325 patients (16%) experiencing complications/reoperation within two years. As seen in Table 4, the most frequent complication was cut-out of the lag screw in the femoral head, with a total of 23 cases (43% of total complications) within two years. Of these, 21/23 cases of cut-out were observed within the first year from surgery. Fifteen cases of femoral head necrosis were observed within two years, 14 of these were seen within the first year. Nine cases of non-union were seen within one year, and no additional cases of non-union were seen after the first year. Six patients were converted to total hip arthroplasty within two years, with three of them being converted within the first year.

### 3.7. Fracture Type

A total of 216 undisplaced (Garden 1/2) and 109 displaced fractures (Garden 3/4) were operated on with DHS during the study period. Displaced fractures resulted in significantly more complications/reoperations than undisplaced fractures within both one year and two years (*p* < 0.001) (Table 1). Displaced/undisplaced fracture type was not associated with mortality rates (Table 1).

### 3.8. Age, CCI Score, Sex, and Operation Time

Unsurprisingly, age was found to be significantly associated with the one-year mortality rate (*p* < 0.001): 83 y (78; 91) for patients deceased <1 year, and 73 y (64; 81) for patients still alive. Age was also found to be significantly associated with the 2-year mortality rate (*p* < 0.001): 83 y (78; 90) for patients deceased <2 years and 71 y (63; 80) for patients still alive. No association between age and complication/reoperation rate was found (Table 1).

Unsurprisingly, the CCI score was found to be significantly associated with the one-year mortality rate, *p* < 0.001; 2 (1; 3) for patients deceased <1 year and 1 (0; 2) for patients still alive. Further, the CCI score was significantly associated with the two-year mortality rate, *p* < 0.001; 2 (1; 3) for patients deceased <2 years and 0 (0; 3) for patients still alive. No association between CCI and complication/reoperation rate was found (Table 1).

Operation time was significantly associated with the two-year mortality rate, *p* < 0.01; 47 min (37; 60) for patients deceased <2 years and 41 min (35; 51) for patients still alive (Table 1). However, operation time was no longer significantly associated with the two-year mortality rate after adjusting for the effect of CCI score, age, and surgical delay, as seen below.

### 3.9. Multivariate Regression Analysis

As seen in Table 1, both CCI score, age, and surgical delay were significantly associated with the mortality rate within 1 year from surgery. CCI score, age, surgical delay, and operation time were significantly associated with the mortality rate 2 years from surgery.

The effects of CCI score and age were adjusted for using a multivariate logistic regression analysis (Table S1). After adjustment, surgical delay remained significantly associated with the one-year mortality rate (*p* = 0.04), and an adjusted odds ratio 1.049 (95%CI 1.002–1.099) was found. However, after adjusting for CCI score, age, and operation time, surgical delay was no longer statistically significantly associated with the two-year mortality rate (*p* = 0.057). Furthermore, operation time was no longer significantly associated with mortality rate two years after surgery (*p* = 0.06).

## 4. Discussion

In this study, we included 325 patients with femoral neck fractures who underwent surgery using DHS to investigate whether surgical delay was associated with mortality rates one and two years after DHS surgery. The National Danish quality guidelines for HF recommend that >90% of HF patients should undergo surgery within 24 h [34]. Over the seven-year study period from 2015–2021, 96% of FNF patients in this study were operated on with DHS within 24 h, with a median surgical delay of 7.9 h. Despite this very short delay to surgery at Regional Hospital Randers, Denmark, we found that surgical delay was significantly associated with both 1-year and 2-year mortality rates.

The second aim of the study was to investigate the association between TAD and the risk of complications/reoperation within 2 years after DHS surgery. We identified relatively few TAD outliers (6/325 with a TAD above 25 mm and 53/325 with a TAD between 20 mm and 25 mm). None of the TAD outliers > 25 mm resulted in complications/reoperation, and only 6 with TAD >20 mm experienced complications or reoperation. Consequently, we did not find an association between TAD and complications/reoperations.

### 4.1. Surgical Delay

The effect of surgical delay on 30-day and 1-year mortality rates remains debated; however, there is a general consensus that surgery should be delayed no more than 48 h [14,15,16,35]. While some studies report no significant association [18,21], others have found that delays exceeding 24 hours are linked to increased mortality [19,20,22]. It has been reported that patients should be operated on within 12 h [36] and that every 10 h of increased delay increases mortality [23]. The literature on the effect of surgical delay and 2-year mortality rates is sparse. Surprisingly, despite the very short delay to surgery in our cohort (<10 h median delay), we found that delay to surgery was significantly associated with the 1-year mortality rates for all (displaced and undisplaced fractures) and showed a significant association with the 2-year mortality rates for undisplaced fractures.

In this single-center study, even surgical delays exceeding 10 h appeared to increase mortality. To better understand the impact of shorter surgical delays—well below 24 h—on mortality in FNF patients treated with DHS surgery, future multi-center studies with larger patient cohorts are needed.

### 4.2. Mortality Rates

High mortality rates after HF have been reported, with 1-year mortality rates reaching approximately 30% for Danish hip fracture patients [9,10]. Compared to these findings, we report a low 1-year mortality rate of just 16% and a 2-year rate of 26%. Our study population, like those in the two referenced cohort studies [9,10], consists of Danish hip fracture patients, making it unlikely that differences in the study population account for our reported 1-year mortality rate. However, the low median surgical delay observed in our study may contribute to this mortality rate, as surgical delay was found to be associated with the 1-year mortality.

### 4.3. Comorbidities

Patient comorbidities and age are known factors influencing mortality rates [10,16]. We also found comorbidity burden, measured by CCI score, and age to be significantly associated with both 1-year and 2-year mortality rates. Remarkably, surgical delay remained significantly associated with the 1-year mortality rates even after adjusting for CCI score and age. However, after adjustment, surgical delay was no longer associated with the 2-year mortality rates.

### 4.4. Complication/Reoperation Rate

When HF are managed with internal fixation, high reoperation rates, ranging from 10–48%, have been reported [24,25,26]. Consistent with these findings, we observed a 1-year complication or reoperation rate of 15% (47 patients), increasing slightly to 16% (53 patients) at two years. Notably, only six additional patients experienced complications or required reoperation after the first year, suggesting that if osteosynthesis survives the first year, the risk of subsequent failure is reduced.

Displaced femoral neck fractures (FNF) carry a higher risk of fixation failure compared to undisplaced fractures when treated with internal fixation [25,35,37]. Consequently, undisplaced FNFs are traditionally managed with internal fixation, whereas displaced FNFs are typically treated with arthroplasty, especially in elderly patients [35,37,38]. As expected, we also found displaced FNF to be associated with a higher rate of complications and reoperations compared to undisplaced fractures.

Because internal fixation of undisplaced FNFs still carries high reoperation rates, recent trends have begun to explore whether arthroplasty might be a more appropriate treatment option in these cases [25,37,39,40,41]. Interestingly, we found that surgical delay was significantly associated with an increased risk of complications or reoperations within two years following internal fixation (DHS-surgery) in patients with undisplaced FNF. However, this association was relatively weak (*p* ≈ 0.046) and should be interpreted with caution. In contrast, no such association was observed in displaced FNFs. Since fracture displacement and intracapsular pressure are known to possibly disrupt the vascular supply to the femoral head [35], it could be hypothesized that early surgical stabilization—by preventing further fracture displacement and reducing intracapsular pressure—may help preserve blood flow and reduce the risk of fixation failure. This mechanism may be less relevant in displaced fractures, where the vascular supply is likely already compromised [35]. Future studies should try to elucidate whether early surgical intervention in undisplaced femoral neck fractures can reduce complication rates after internal fixation.

### 4.5. Implant Placement (TAD)

The most common complication after internal fixation with lag screws is a cut-out of the screw in the femoral head [27]. Adding to this, we report cut-out as the most frequent complication following DHS surgery in FNF, with 23 of 53 complications (43%) occurring within 2 years. A TAD >25 mm was identified as the critical cut-off point for the risk of cut-out in 1995 [28] and has been widely incorporated in clinical practice. Since TAD as a risk factor has been debated, and various cut-off points, including 19.9 mm, 27 mm, and 38.8 mm, have been reported [27,29,42,43]. Some studies have found that TAD alone cannot predict the risk of cut-out [44,45]. Adding to this, we report no association between TAD and the risk of complications/reoperation in FNF patients within 2 years of DHS surgery. Notably, none of the TAD outliers > 25 mm resulted in complications or reoperation. Only 59/325 patients in this study had a TAD >20 mm, just 6 of these experienced complications, and no correlation with the risk of cut-out with TAD >20 mm was found. Although this finding is interesting, it should be emphasized that only 6 patients in our study had a TAD >25 mm. Consequently, it is not possible to draw firm conclusions regarding the association between TAD and the risk of complications/reoperation. Further studies with larger samples of TAD outliers (>20 or >25 mm) are needed to fully clarify this relationship.

### 4.6. Limitations

Most electronic patient files did not report information on smoking and alcohol habits. Therefore, only an indirect measure could be obtained when patients suffered from liver cirrhosis based on alcohol consumption, since liver cirrhosis is covered in the CCI score. We were therefore not able to fully adjust for smoking and alcohol habits.

Only data from the electronic patient files and radiographs within the Central Denmark Region were included. Therefore, if patients migrated out of the region within the 2 years of follow-up, no reports on complications or reoperation would exist in the journal or the radiographic material. It is therefore possible that complications/reoperations are underreported. However, migration in our study population seems less likely. Potential migration to another Danish region would not affect the estimated mortality rates, as this information is automatically updated nationwide.

Information on the operation start was obtained from the Central Denmark Region’s Business Intelligence portal. The validity of this portal was not assessed. It contains data recorded by clinicians in electronic patient journals, which may introduce some information bias. This could result in non-differential inaccuracies in estimating the operation start.

We have a rather small sample size (n = 325) and only 53 patients experienced complications. Some trends may be masked or hidden due to the limited population group.

In general, the retrospective nature of the study poses a limitation due to the potential underreporting of complications. However, in this study, complications were defined based on radiographic findings, reoperations, and mortality. Thus, these criteria are likely more reliable than those used in other retrospective studies, where complications such as systemic infections at home or in nursing homes are more likely to go underreported.

## 5. Conclusions

Despite minimal surgical delay in the 325 femoral neck fracture (FNF) patients osteosynthesized with DHS—96% were operated on within 24 h—surgical delay was significantly associated with the 1-year mortality. In undisplaced fractures, surgical delay was also significantly associated with complication and reoperation rates within 2 years. No correlation between tip–apex distance (TAD) and complication/reoperation rates was found; however, only 59 of 325 patients had a TAD >20 mm, limiting the ability to draw firm conclusions.

These findings suggest that even relatively short surgical delays may affect outcomes, underscoring the importance of prioritizing early surgery in clinical workflows. Future multi-center studies with larger cohorts are needed to explore whether earlier surgical intervention can reduce both mortality and—particularly in undisplaced fractures—complication rates, and to clarify the clinical relevance of TAD thresholds in relation to reoperation risk.

## Figures and Tables

**Table 1:** Overview of the investigated parameters and their influence on the risk of complications within 1 and 2 years (top columns) and on the risk of 1-year and 2-year mortality (bottom columns). The type of fracture (displaced/undisplaced) was significantly associated with the risk of complications/reoperation within 1 and 2 years, with displaced fractures having significantly more reoperations than undisplaced fractures. Displaced/undisplaced fracture type did not influence mortality rates. The CCI score, age, and surgical delay were found to have a significant association with the 1-year and 2-year mortality rates, while operation time had a significant association with the 2-year mortality. Statistical significance is highlighted with bold.

	Complications 1 Year			Complications 2 Years		
	None	<1 year	*p*-value	None	<2 years	*p*-value
Undisplaced fractures (n)	196	20	**<0.001**	194	22	**<0.001**
Displaced fractures (n)	82	27		78	31	
CCI score	1 (0; 2)	1 (0; 2)	0.77	1 (0; 2)	1 (0; 2)	0.74
Age (years)	75 (66; 83)	70 (63; 80)	0.22	75 (66; 83)	69 (63; 80)	0.05
Female (n)	166	32	0.27	162	36	0.25
Male (n)	112	15		110	17	
Operation time (min)	46 (36; 56)	49 (36; 61)	0.56	45 (37; 56)	51 (37; 65)	0.11
Surgical delay (h)	7.9 (5.1; 14)	8.7 (4.8; 14)	0.77	7.8 (5.1; 14)	8.9 (4.9; 14)	0.83
TAD (mm)	16.3 (14; 19)	16.7 (14; 19)	0.56	16.3 (14; 19)	16.6 (14; 19)	0.99
	1-year Mortality			2-year Mortality		
	Still alive	Deceased < 1 year	*p*-value	Still alive	Deceased < 2 years	*p*-value
Undisplaced fractures (n)	191	25	0.1	165	51	0.14
Displaced fractures (n)	89	20		75	34	
CCI score	1 (0; 2)	2 (1; 3)	**<0.001**	0 (0; 3)	2 (1; 2)	**<0.001**
Age (years)	73 (64; 81)	83 (78; 91)	**<0.001**	71 (63; 80)	83 (75; 90)	**<0.001**
Female (n)	167	31	0.83	151	47	0.16
Male (n)	106	21		88	39	
Operation time (min)	43 (36; 54)	46 (37; 57)	0.15	41 (35; 51)	47 (37; 60)	**<0.001**
Surgical delay (h)	7.5 (4.7; 13.9)	10.9 (6.9; 17.1)	**<0.01**	7.5 (4.6; 13.8)	10.3 (6.4; 15.2)	**<0.01**
TAD (mm)	16.3 (14; 19)	16.4 (14; 24)	0.6	16.3 (14; 19)	16.3 (14; 19)	0.6

**Table 2:** Subgroup analysis of the association among surgical delay, fracture type, and complication/reoperation rate. No effect of surgical delay on the 1-year complication rate was found between undisplaced fractures (Garden 1/2; *p* = 0.05) and displaced fractures (Garden 3/4; *p* = 0.33). However, a significant association between surgical delay and the risk of complications/reoperation within 2 years was found for undisplaced fractures (*p* = 0.0464). Median surgical delay was 13.54 h (7.5; 15.8) for patients with complications and 7.6 h (5.1; 14.7) for patients without complications. No association was found for displaced fractures (*p* = 0.31). Statistical significance is highlighted with bold.

	Surgical Delay					
	No complications < 1 year	Complications < 1 year	*p*-value	No complications < 2 years	Complications < 2 years	*p*-value
All	7.85 (5.1; 14.4)	8.7 (4.8;1 4.4)	0.77	7.83 (5.1; 14.6)	8.9 (4.9; 14)	0.83
Garden 1/2	7.6 (5.1; 13.6)	13.7 (6.5; 17.4)	0.054	7.6 (5.1; 14.7)	13.54 (7.5; 15.8)	**0.046**
Garden 3/4	8.3 (4.7; 14.4)	6.7 (4.5; 12)	0.33	8.3 (4.7; 14.4)	6.7 (4.5; 12)	0.31

**Table 3:** Overview of mortality rates. The overall mean 1-year mortality rate for the study period was 16%, and 26% for the 2-year mortality rate. The lowest 1-year mortality rate was observed in 2021 (10.2%), and the highest was seen in 2018 (24.5%). The lowest 2-year mortality rate was observed in 2021 (18.6%), and the highest was recorded in 2018 (34.7%).

Mortality					
	Deceased < 1 year (n)	(%)	Deceased < 2 year (n)	(%)	Total number of patients (n)
**Year**					
2015	5	17.9	6	21.4	28
2016	6	19.4	8	25.8	31
2017	6	15.4	11	28.2	39
2018	12	24.5	17	34.7	49
2019	5	10.2	11	22.5	49
2020	12	17.1	22	31.4	70
2021	6	10.2	11	18.6	59
Total	52	16	86	26	325

**Table 4:** Overview of complications. The first number in each square represents the total number of complications within 1 year from surgery. The second number represents the total number of complications after the first year from surgery, but within 2 years from surgery. A total of 47 complications/reoperations were found within 1 year, and 53 cases within 2 years. It should be noted that only an additional six patients experienced complications after the first year, but within 2 years of surgery. The most frequent complication was cut-out of the lag screw in the femoral head, with 21 cases within 1 year and an additional 2 cases after the first year but within 2 years, resulting in a total of 23 cases within 2 years.

	2015	2016	2017	2018	2019	2020	2021	Total Study Period
Complications (n < 1y; n < 2y)								
Cut-out	2; 0	1; 0	3; 0	0; 1	5; 0	7; 1	3; 0	21; 2
Necrosis of femoral head	3; 0	3; 0	1; 0	2; 0	1; 0	2; 0	2; 1	14; 1
Non-union	0; 0	1; 0	2; 0	2; 0	1; 0	1; 0	2; 0	9; 0
Pain, artrosis, reduced offset	0; 1	0; 0	1; 0	0; 0	1; 0	0; 0	1; 2	3; 3
Year total	5; 1	5; 0	7; 0	4; 1	8; 0	10; 1	8; 3	47; 6

## Data Availability

The data that support the findings of this study are not openly available due to reasons of sensitivity. However, the data are available from the authors upon reasonable request and with permission from the Central Denmark Region Ethical Committee. The data are stored in controlled-access repositories at Central Denmark Region.

## Supplementary Materials

The following supporting information can be downloaded at: https://www.mdpi.com/article/10.3390/jcm14144991/s1, Table S1: Logistic multivariate regression analysis.

## Abbreviations

The following abbreviations are used in this manuscript:

FNF	Femoral neck fracture
DHS	Dynamic hip screw
TAD	Tip–apex distance
Y	Year
HF	Hip fractures
IMN	Intramedullary nailing
CCI	Charlson Comorbidity Index
EPJ	Electronic patient file system
