# Supplementary material for: Are Tip–Apex Distance and Surgical Delay Associated with Increased Risk of Complications and Mortality Within the First Two Years After Surgery for Femoral Neck Fractures?
Source: J Clin Med. 2025 Jul 15;14(14):4991. doi: 10.3390/jcm14144991 (PMC12294839; doi:10.3390/jcm14144991)
Supplement: Supplementary file 1 [file jcm-14-04991-s001.zip › jcm-3610648-supplementary.pdf]

Supplementary Material

**Dependent variable:**	**Deceased within 1 year**				
Prob >chi2 0.000		Odds ratio	95% CI	z-value	*p*-value
	Independet variables:				
	CCI	1.35	1.15 - 1.59	3.64	**<0.01**
	Age	1.11	1.06 - 1.15	5.44	**<0.01**
	Surgical delay (hours)	1.05	1.002 - 1.1	2.04	**0.041**

**Dependent variable:**	**Deceased within 2 years**				
Prob >chi2 0.000		Odds ratio	95% CI	z-value	*p*-value
	Independet variables:				
	CCI	1.39	1.19 - 1.63	4.15	**<0.01**
	Age	1.10	1.07 - 1.14	6.06	**<0.01**
	Surgical delay (hours)	1.0001	0.99 - 1.001	2.04	0.057
	Operation time	0.99	0.97 - 1.001	-1.85	0.064

Table S1 Logistic multivariate regression analysis.

A logistic multivariate regression analysis was made to adjust for the effect of CC score, age, and surgical delay on the 1-year mortality rate. After adjusting, all 3 parameters remained significantly correlated with the 1-year mortality rate. An additional logistic multivariate regression analysis was made to adjust for the effect of CCI score, age, surgical delay, and operation time on the 2-year mortality rate. After adjusting only CCI score and age remained significantly correlated with the 2-year mortality rate.
